# Association between atypical swallowing and malocclusions: a systematic review

**DOI:** 10.1590/2177-6709.27.6.e2221285.oar

**Published:** 2023-03-27

**Authors:** Flávio Magno GONÇALVES, Karinna Veríssimo Meira TAVEIRA, Cristiano Miranda de ARAUJO, Glória Maria Nogueira Cortz RAVAZZI, Odilon GUARIZA, Bianca Simone ZEIGELBOIM, Rosane Sampaio SANTOS, Jose STECHMAN

**Affiliations:** 1 Universidade Tuiuti do Paraná, Programa de Pós-Graduação em Distúrbios da Comunicação (Curitiba/PR, Brazil).; 2 Universidade Federal do Rio Grande do Norte, Programa Associado de Pós-graduação em Fonoaudiologia (Natal/RN, Brazil).; 3 Pontifícia Universidade Católica do Paraná, Departamento de Ortodontia (Curitiba/PR, Brazil).; 4 Núcleo de Estudo Avançado em Revisão Sistemática e Meta-análise (NARSM).

**Keywords:** Deglutition disorders, Deglutition, Malocclusion, Review

## Abstract

**Objective:**

This systematic review aims to answer the following focus question: “*Is there an association between atypical swallowing and malocclusions?”.*

**Methods:**

Appropriate word combinations were chosen and tailored specifically for each of the following electronic databases: EMBASE, Latin American and Caribbean Literature in Health Sciences (LILACS), LIVIVO, PubMed/Medline, Scopus, Web of Science, and gray literature, without any restrictions, up to February 2021. According to the selection criteria, only cross-sectional studies were included. The following inclusion criteria were considered: a sample composed of children, adolescents, and adults; patients clinically diagnosed with atypical swallowing; patients with normal swallowing; and outcome of interest of atypical swallowing in patients with malocclusion. The data consisted of study characteristics, sample characteristics, results, and conclusion of each study. The risk of bias was assessed using the JBI Critical Appraisal Checklist for Analytical Cross-Sectional Studies, and the certainty of evidence was assessed using the GRADE tool.

**Results:**

4,750 articles were identified. After a two-step selection, four studies were included. A higher frequency of distal occlusion, extreme maxillary overhang, and open bite was related to swallowing disorders; most studies pointed to posterior crossbite as a malocclusion more associated with atypical swallowing. All studies had a moderate to high risk of bias, and the certainty of evidence was very low.

**Conclusion:**

The results indicate that atypical swallowing is associated with malocclusions and that posterior crossbite is the main malocclusion found, but only in the young population (3-11 years).

**Registration:**

PROSPERO (42020215203).

## RESUMO

**Objetivo:**

A presente revisão sistemática tem como objetivo responder à seguinte questão focal: *“Existe associação entre deglutição atípica e más oclusões?”*.

**Métodos:**

Combinações de palavras e truncamentos apropriados foram adaptados para as bases de dados eletrônicas: EMBASE, Literatura Latino-Americana e do Caribe em Ciências da Saúde (LILACS), LIVIVO, PubMed/Medline, Scopus e Web of Science e a literatura cinzenta, sem qualquer restrição, até fevereiro de 2021. Os critérios de inclusão foram: estudos transversais; amostra composta por crianças, adolescentes e adultos; pacientes com diagnóstico clínico de deglutição atípica; pacientes com deglutição normal, e o desfecho de interesse foi deglutição atípica em pacientes com má oclusão. Os dados extraídos de cada estudo foram características do estudo, da amostra e a conclusão. O risco de viés foi avaliado usando a Lista de verificação de avaliação crítica da JBI para estudos transversais analíticos, e a certeza das evidências foi avaliada usando a ferramenta GRADE.

**Resultados:**

Foram identificados 4.750 artigos. Após uma seleção em duas fases, quatro estudos foram incluídos. Maior frequência de disto-oclusão, protuberância maxilar extrema e mordida aberta foram relacionadas a distúrbios da deglutição, com a maioria dos estudos apontando para mordida cruzada posterior como a má oclusão mais associada à deglutição atípica. Todos os estudos tiveram um risco de viés moderado a alto, e a certeza das evidências foi considerada muito baixa.

**Conclusão:**

Os resultados indicam que a deglutição atípica está associada à mordida cruzada posterior apenas na população jovem de 3 a 11 anos.

## INTRODUCTION

Atypical swallowing is a myofunctional problem characterized by a postural change in the tongue during the swallowing process.^1^ It has a multifactorial etiology and involves non-functional habits, genetics, mouth breathing, and allergic processes.^1^ ^-^ ^3^ Initially, physiological swallowing, also called visceral swallowing or infant swallowing, is the lack of activation of masticatory muscles and use of the orbicularis oris muscle, with lingual interposition between the alveolar crests of the upper and lower incisors, thus generating a negative intraoral pressure, which in turn enables the functions of sucking and swallowing. Atypical swallowing happens if there is no complete maturation from the action of infant swallowing to a more conscious and voluntary action.^2^

Atypical swallowing is considered a risk factor mainly for anterior open bite and posterior crossbite, affecting the development of occlusion by neuro-muscular imbalance. Non-functional habits, whenever present, also directly affect the development of swallowing and occlusion, especially that related to anterior open bite. The patient’s history is also important, and should not be disregarded. The treatment of atypical swallowing (myofunctional therapy) and orthodontic treatment are closely related, as both processes need mutual support for a better outcome and stability.^4^ ^,^ ^5^ Thus, a balance of the entire stomatognathic system is necessary for a proper functioning of all functions this system performs.

The relationship between atypical swallowing and malocclusion is widely discussed in the literature, but there is no systematic review that evaluates this association including only studies with adequate diagnostic methods for atypical swallowing. Therefore, the objective of this systematic review is to answer the following focus question: *“Is there an association between atypical swallowing and malocclusions?”.*

## METHOD

### PROTOCOL

This systematic review was carried out in accordance with the guidelines of PRISMA ^6^ (Preferred Reporting Items for Systematic Review and Meta-Analysis).

### ELIGIBILITY CRITERIA

To consider the eligibility of studies for inclusion/exclusion from this review, the acronym “PECOS” was used.

» Population (P): sample composed of children, adolescents, and adults.
» Exposure (E): patients clinically diagnosed with atypical swallowing.
» Comparison (C): patients with normal swallowing.
» Outcomes (O): the outcome of interest of atypical swallowing in patients with malocclusion.
» Study design (S): observational studies.

Studies in which the sample consisted of patients diagnosed with atypical swallowing were included. Studies that evaluated malocclusion as an independent variable and the diagnosis of atypical swallowing as an outcome were also included, and their results were described separately. The assessment of malocclusion and atypical swallowing was by clinical assessment. Mandatory comparison to a control group: normal swallowing or normal occlusion. Observational, case-control, cohort, and cross-sectional studies were included. There was no discrimination regarding ethnicity, gender, age, language, or year of publication.

The following exclusion criteria were applied: 1) patients that had undergone previous or undergoes current orthodontic treatment, patients with neurological disorders or craniofacial deformities; 2) patients not clinically diagnosed with malocclusion or atypical swallowing; 3) studies with no control group; 4) studies that did not assess malocclusion and atypical swallowing as an outcome; 5) reviews, letters, books, conference abstracts, case reports, case series, opinion articles, technical articles, guidelines, randomized or non-randomized methods, and clinical trials; 6) the full text of the study was not available. 

### INFORMATION SOURCES AND SEARCH STRATEGY

Appropriate word combinations and truncations were adapted for each of the six electronic databases chosen as information sources: EMBASE, Latin American and Caribbean Literature on Health Sciences (LILACS), LIVIVO, PubMed/Medline, Scopus, and Web of Science. In addition, gray literature was also a source of information through Google Scholar, Open Gray, and ProQuest Dissertation and Thesis (Appendix 1). Manual reference consultation was performed from the references section of all studies included and with experts, in order to improve the search results and following the recommendations of Greenhalgh and Peacock.^7^ Searches on electronic databases and gray literature were performed on July 10, 2020 and updated on February 26, 2021. References were managed, and duplicate studies were removed using appropriate software (EndNote^®^ X7 Thomson Reuters, Philadelphia, PA).

### SELECTION OF STUDIES

The selection of studies was carried out in two steps. In the first step, two reviewers (FMG and KVTM) independently reviewed the titles and abstracts of all studies. All articles that did not meet the established eligibility criteria were excluded at this step. In the second step, the same reviewers independently read the full text of the studies selected in the first step. Whenever there was any disagreement and the lack of consensus persisted even after discussion, a third reviewer (CMA) provided the final decision.

To facilitate independent reading in both steps, the Rayyan^®^ website was used (http://rayyan.qcri.org), where reviewers are blind in all assessments and a third team member acts as moderator.

### DATA COLLECTION PROCESS

Two reviewers (FMG and KVMT) independently collected information from the studies included. The information was discussed with two experts in the field. The data collected consisted of study characteristics (author, year of publication, country, title, and study design), sample characteristics (sample size, control group, form of diagnosis of malocclusion and atypical swallowing), outcomes, and results (Table 1). When data were missing or incomplete, attempts were made to contact the authors for important unpublished information. The authors were contacted by email for three consecutive weeks whenever more information was needed.

**Table 1:** Summary of the characteristics of the studies included (n=4).

Author, Year, Country	Objective	Sample size, characteristics and exposition	Malocclusion studied and form of diagnosis	Diagnosis of atypical swallowing	Outcome of interest	Prevalence ratio* (95% CI)
Melink et al.^13^, 2010, Slovenia	Find an association between posterior crossbite, sucking habits, orofacial functions, and otorhinolaryngological findings	30 children with posterior crossbite (13 boys, 17 girls, mean age: 5.5 years, range: 3.6-7.2 years) 30 children without posterior crossbite (17 boys, 13 girls; mean age 5.9 years, range 5.4-6.7 years)	Posterior crossbite diagnosed by clinical evaluation	Evaluated by the method suggested by Melsen et al.^12^, 1979	Crossbite group: 6 (22%) with atypical swallowing Group without crossbite: 2 (8%) with atypical swallowing	3.00 [0.66, 13.69]*
Melsen et al.^12^, 1979, Denmark	Analyze the relationship of sucking habits, swallowing pattern, and prevalence of malocclusions	A total of 723 children were evaluated (366 boys, 357 girls) aged 10-11 years; of which 313 children with atypical swallowing and 399 children with normal swallowing	Authors classified all malocclusions in the sample, including posterior crossbite. Clinical evaluation and molding	Clinical evaluation and palpation of the masseter and temporal muscles	Of the 313 children with atypical swallowing, 44 had a diagnosis of posterior crossbite. In the group of 399 children with normal swallowing, only 42 had posterior crossbite	1.335 [0.85, 2.09]*
Ovsenik et al.^10^, 2009, Slovenia	To investigate the prevalence of crossbite in 5-year-old Slovenian preschoolers and its relationship with atypical swallowing habits and patterns at 3, 4 and 5 years of age	243 children (119 boys, 124 girls), assessed at ages 3, 4 and 5 years. 206 children without crossbite and 37 children with posterior crossbite	Posterior crossbite diagnosed by clinical evaluation and obtaining plaster models	Assessed by the method suggested by Melsen et al.^12^, 1979	Presence of atypical swallowing in: 206 children without crossbite at 5 years of age: 35% (n=72) 37 children with posterior crossbite at age 5 years: 63% (n=23)	2.27 [1.27, 4.04]*
Ovsenik et al.^11^ 2014, Slovenia	To assess the prevalence of swallowing in patients with and without Unilateral Posterior Crossbite using ultrasound examination	23 children with unilateral posterior crossbite (13 girls and 10 boys), aged 4.1-6.6 years). Average age 5.4 ± 0.8 years 22 children without unilateral posterior crossbite (10 girls and 12 boys) aged 5.7-6.7 years. Average age 6.1 ± 0.3 years.	Clinically evaluated by a calibrated orthodontist	Swallowing pattern was determined according to the method described by Peng et al.^16^, 2003. Through ultrasound examination	83% (n=22) of children with unilateral posterior crossbite had visceral swallowing. Only 36% (n=8) of children without unilateral posterior crossbite presented this swallowing type (*p*= 0.002)	1.78 [1.30, 4.04]*

* values calculated by the authors.

### RISK OF BIAS IN INDIVIDUAL STUDIES

The studies were assessed as for methodological quality using the JBI Critical Appraisal Checklist for Analytical Cross Sectional Studies.^8^ Two reviewers (FMG and KVMT) performed a risk of bias assessment separately and judged the articles included, marking each assessment criterion with “yes,” “no,” “uncertain,” and “not applicable.” The risk of bias was high when the study reached 49% of “Yes”; moderate when the study reached 50% to 69% of “Yes”; and low when the study reached more than 70% of “Yes.” Whenever necessary, disagreements were resolved through discussion with a third reviewer (CMA). The Revman 5.4^®^ software (Review Manager 5.4; The Cochrane Collaboration) was used to generate figures.

### MEASUREMENT SUMMARY

The number of events and the sample size were collected from each study to calculate the measure of association. For the cross-sectional studies included, the prevalence ratio was calculated with the 95% confidence intervals (CI). 

### PUBLICATION BIAS ASSESSMENT

Whenever possible (n > 10), publication bias is investigated using funnel plots. However, this assessment was not possible in the present study. A broad search strategy in electronic databases and gray literature, besides consulting the expert as for unpublished articles, was carried out in order to reduce the risk of publication bias.

### RELIANCE ON CUMULATIVE EVIDENCE

The results were analyzed using GRADE^®^ (Classification of Recommendations, Evaluation, Development and Evaluation, https: //gradepro.org/), which is a quality scoring system.^9^ Two reviewers judged the following aspects: risk of bias, inconsistency, indirect evidence, imprecision, and publication bias. The level of evidence was high, moderate, low, or very low. Disagreements were resolved by consensus, and a third reviewer was consulted whenever necessary.

## RESULTS

### SELECTION OF STUDIES

**Figure 1:**
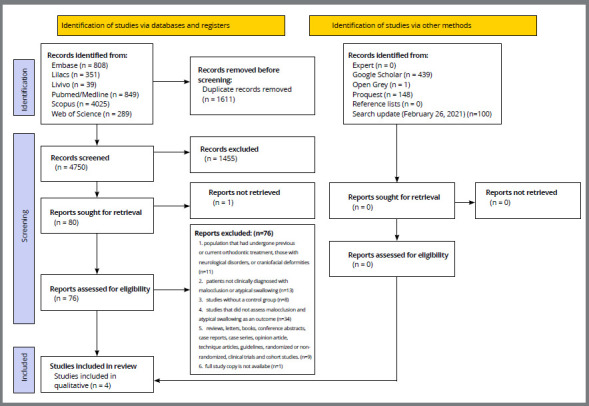
Flow diagram of the literature search and the selection criteria.

Source: Page MJ, McKenzie JE, Bossuyt PM, Boutron I, Hoffmann TC, Mulrow CD, et al. The PRISMA 2020 statement: an updated guideline for reporting systematic reviews. BMJ 2021;372:n71. doi: 10.1136/bmj.n71. For more information, visit: http://www.prisma-statement.org/

A total of 6,361 references were retrieved in the six electronic databases, remaining 4,750 references after the removal of duplicates. After reading the titles and abstracts (step 1), 80 articles were selected for full reading (step 2), of which 76 were excluded (Appendix 2). Following a throughout article review, four articles were later included (Fig 1). No additional articles were included from the reference lists, gray literature, and consultations with experts.

### STUDY CHARACTERISTICS

Four cross-sectional studies were included.^10^ ^-^ ^13^ They were published between 1979 and 2010 and carried out in Slovenia^10^ ^,^ ^11^ ^,^ ^13^ and Denmark.^12^ The age of the individuals included in the selected articles ranged from 3^10^ to 11 years,^12^ and the sample size ranged from 45^11^ to 723^12^ individuals. 

One study evaluated the presence of all malocclusions.^12^ Three studies assessed exclusively posterior crossbite,^10^ ^,^ ^11^ ^,^ ^13^ and only one of these studies classified posterior crossbite as unilateral.^11^

The evaluation of occlusion was performed using clinical analysis by a trained professional^10^ ^-^ ^13^ using plaster models.^10^ ^,^ ^12^ Swallowing was assessed using only clinical assessment. The method used for this assessment was developed by Melsen et al.^12^ (1979) and Ovsenik et al.^11^ (2014). 

### RISK OF BIAS IN STUDIES

The risk of bias in individual studies revealed three studies as having a moderate risk^10^ ^,^ ^11^ ^,^ ^13^ and one study a high risk of bias.^12^

**Figure 2:**
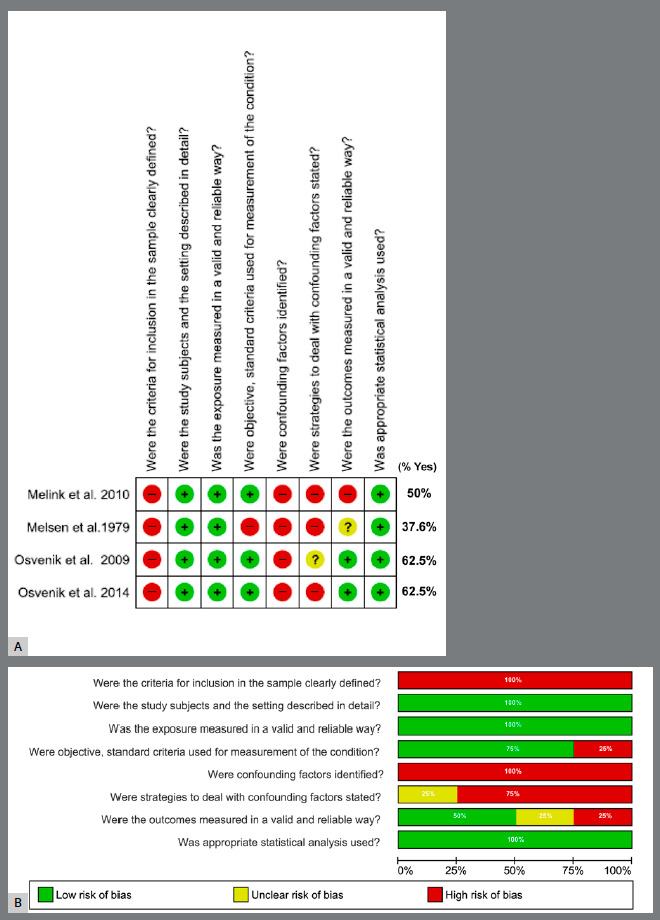
Summary of risk of bias assessed by Joanna Briggs Institute Critical Appraisal Checklist for Analytical Cross-Sectional Studies: author’s judgments for each study included (generated using the software Review Manager 5.4, The Cochrane Collaboration).

The methodological limitations in all studies included in this review were related to deficient reports of sample inclusion and exclusion criteria, confounding factors, and control strategy of these factors. All studies were classified as having a “low risk” of bias regarding the description of participating subjects, environments, valid and reliable exposure analyses, and appropriate statistical analyses. Figures 2A and 2B summarize the evaluations obtained by the JBI tool.

### RESULTS OF INDIVIDUAL STUDIES

Melink et al.^13^ (2010) aimed to analyze posterior crossbite in the period of primary dentition and its relationship with sucking habits, irregular orofacial functions, and otorhinolaryngological findings. The authors found a prevalence of 22% in children with atypical swallowing crossbite and 8% in children in a group without this malocclusion.

Evaluating 725 Danish children, which is the largest sample among the studies included in this review, Melsen et al.^12^ (1979)reported on sucking habits, swallowing patterns, and prevalence of malocclusions. Compared to the group of children with normal swallowing, children with impaired swallowing had an increased frequency of distal occlusion, extreme maxillary projection, and open bite. Special attention should be given to non-nutritive sucking habits, since they may influence the development of swallowing and occlusion.

Ovsenik et al.^10^ (2009) analyzed issues in orofacial functions of 243 five-year-old children and the association with posterior crossbite. The authors found that the atypical pattern of swallowing increased over time in children with crossbite, and that in children without posterior crossbite this atypical pattern had a statistically significant decrease. 

Ovsenik et al.^11^ (2014), using ultrasound equipment, found an atypical swallowing pattern in 83% of children with unilateral posterior crossbite, a statistically significant number, compared with the 36% rate of atypical swallowing pattern in children with normal occlusion. 

### REPORTING BIAS

As it was impossible to carry out an evaluation using the funnel chart (n**˂**10) to reduce the probability of publication bias, a wide search was carried out in several databases, including a database in a language other than English (LILACS), and in gray literature.

### RELIANCE ON CUMULATIVE EVIDENCE

The overall certainty of evidence identified using GRADE^9^ was very low, due to the following reasons: high risk of bias was considered “serious”, since no study reported exclusion factors, confounding variables, unreliable measurements of outcomes, and inaccuracy related to small sample sizes and number of events, and indirect evidence indicated that there was no association with the adolescent and adult populations. There was no publication bias, as there was an effort to search all the literature available on the subject, including gray literature. Furthermore, a potential conflict of interest of the studies included was not detected (Table 2).

**Table 2:** Analysis of information quality through GRADE. **Research question:** *“Is there an association between atypical swallowing and malocclusions?”*

Certainty assessment							№ of patients		Effects		Overall certainty of evidence
№ of studies	Study design	Risk of bias	Inconsistency	Indirect evidence	Imprecision	Publication bias	Posterior crossbite	Placebo	Relative (95% CI)	Absolute (95% CI)	
3	Observational study	Serious^a^	No serious	Serious^b^	Serious^c^	none	48/90 (53.3%)	82/258 (31.8%)	RR 1.98 (1.49 to 2.63)	311 more per 1.000 (from 156 more to 518 more)	⨁◯◯◯ VERY LOW

CI = Confidence interval; RR = Risk ratio.

Explanations:

^a^ Exclusion factors were not described, showed confounding factors and outcome measures were unreliable.

^b^ There was no association with the population of adolescents and adults.

^c^ The sample size or the number of events does not meet the optimal information (Cochrane handbook, Chapter 14).

## DISCUSSION

This systematic review investigates the available evidence on the association between atypical swallowing and malocclusions. This relationship is widely studied, but the cause and effect relationship is still controversial.^14^ Due to the longitudinal nature of this relationship and the observational nature of the studies, it was possible to determine a greater association between posterior crossbite and atypical swallowing. This is an interesting fact, considering that open bite is generally related to atypical swallowing both in the literature and in the clinic.^2^ ^,^ ^15^ This data corroborates that described in the literature.^2^ ^,^ ^10^ ^-^ ^17^

It is important to highlight the results found by Osvenik et al.^11^ (2014). The authors reported that atypical swallowing has the same prevalence in children up to three years of age, regardless of whether they have malocclusion or not. However, advancing age of patients who presented posterior crossbite had an increase in the condition of atypical swallowing, and those who did not have this malocclusion had a significant low presence of atypical swallowing.^11^ This data makes the present authors consider that posterior crossbite and atypical swallowing are closely related, and that the treatment for malocclusion and atypical swallowing has better results in the long term when it begins early.^3^

Atypical swallowing, regardless of age, happens both in children and adults. During the study selection phase, some studies with a population of adolescents and/or adults did not meet all eligibility criteria, because this population had undergone prior dental (orthodontic) treatment or because they had an associated comorbidity.^18^

Some studies suggest that anterior open bite is also associated with the habit of atypical swallowing. The incorrect posture of the tongue during the swallowing movement affects and perpetuates the presence of this malocclusion.^12^ ^,^ ^15^ ^,^ ^17^ However, in the case of open bite, it is important for the professional to assess the history of non-functional habits and whether their presence significantly affects the type of swallowing.^12^ However, this type of malocclusion was little discussed in this study due to the lack of an adequate methodology for primary sources. To carry out a review of associations, the presence of a control group is necessary, thus demonstrating the need for adequate clinical research designs to analyze this topic.

In this systematic review it was possible to observe more data on posterior crossbite, counting three studies^10^ ^,^ ^11^ ^,^ ^13^, due to the methodological heterogeneity between them. A study included the outcome of assessed malocclusion in a population with atypical swallowing,^12^ and in this same study the authors observed that non-functional sucking habits, even of short duration, may have an indirect effect on the swallowing pattern and a direct effect on occlusion development.

There is a variety of techniques and instruments for the assessment and diagnosis of atypical swallowing.^14^ Only Osvenik et al.^11^ used ultrasound equipment to assess swallowing. It was possible to observe by this non-invasive technique, the duration, amplitude, and speed of the tongue’s movements during swallowing. All other studies performed only the clinical assessment described by Melsen et al.^12^ (1979) without following internationally validated assessment protocols. These protocols are usually not applied by a professional capable of performing a diagnosis of swallowing disorders, for example speech therapists.^19^

It is noteworthy that the certainty of evidence was rated very low by the GRADE^®^ system. The explanation for this is the risk of bias of the studies included, unclear exclusion criteria, uncontrolled confounding factors, a population comprising of only children, and geographical boundaries (including only two countries), and the low number of studies. There are some limitations to this review: the use of non-validated tools to assess atypical swallowing, assessments based solely on clinical assessment, and few articles that meet the eligibility criteria. Therefore, further studies need a greater methodological rigor and should perform a greater control of confounding factors, as the current literature does not yet report a high certainty of evidence on this topic.

Additional studies are recommended using standardized and validated swallow assessment protocols.

## CONCLUSIONS

This systematic review investigated the evidence available on the association between atypical swallowing and malocclusions. Based on the current literature available and on eligibility criteria established for this systematic review, atypical swallowing is associated with malocclusions. Posterior crossbite is the main malocclusion found. The results are relevant only for the young population (3-11 years), and it is not possible to generalize them to other age groups (adolescents and adults). However, based on the level of certainty, the results should be evaluated with caution. Further studies with better methodological quality are thus suggested.

## Appendix 1: Database search strategy.

[Table t1a]

**Table t1a:**

Database	Search (July 10^th^ 2020; updated on February 26^th^, 2020)
Cochrane Library	(“Deglutition Disorders” OR “Deglutition Disorder” OR “Swallowing” OR “swallowed” OR “swallowings” OR “swallowable” OR “swallower” OR “swallowers” OR “swallows” OR “swallow” OR “Swallowing Disorders” OR “Swallowing Disorder” OR “atypical swallowing” OR “atypical deglutition” OR “Myofunctional”) AND (“Malocclusions” OR “malocclusion” OR “dental position” OR “dental positions” OR “tooth crowding” OR “crossbite” OR “crossbites” OR “cross-bite” OR “cross-bites” OR “cross-bite” OR “cross-bites” OR “open-bite” OR “openbite” OR “open-bite” OR “deep bite” OR “Overbites” OR “overbite” OR “deep bite” OR “deep bites” OR “deep bite” OR “deep bites” OR “Over Bite” OR “Over Bites” OR “Angle’s Classification” OR “Angle Classification” OR “Angles Classification” OR “angle class I” OR “angle class II” OR “Angle Class III” OR “Class I” OR “Class II” OR “Class III” OR “malocclusion, angle class III” OR “angle class III malocclusion” OR “skeletal class III malocclusion” OR “class III malocclusion” OR “maxillofacial development” OR “angle class II” OR “angle class II malocclusion” OR “skeletal class II malocclusion” OR “class II malocclusion” OR “angle class I” OR “angle class I malocclusion” OR “skeletal class I malocclusion” OR “class I malocclusion”)
Embase	(“Deglutition Disorders” OR “Deglutition Disorder” OR “Swallowing” OR “swallowed” OR “swallowings” OR “swallowable” OR “swallower” OR “swallowers” OR “swallows” OR “swallow” OR “Swallowing Disorders” OR “Swallowing Disorder” OR “atypical swallowing” OR “atypical deglutition” OR “Myofunctional”) AND (“Malocclusions” OR “malocclusion” OR “dental position” OR “dental positions” OR “tooth crowding” OR “crossbite” OR “crossbites” OR “cross-bite” OR “cross-bites” OR “cross-bite” OR “cross-bites” OR “open-bite” OR “openbite” OR “open-bite” OR “deep bite” OR “Overbites” OR “overbite” OR “deep bite” OR “deep bites” OR “deep bite” OR “deep bites” OR “Over Bite” OR “Over Bites” OR “Angle’s Classification” OR “Angle Classification” OR “Angles Classification” OR “angle class I” OR “angle class II” OR “Angle Class III” OR “Class I” OR “Class II” OR “Class III” OR “malocclusion, angle class III” OR “angle class III malocclusion” OR “skeletal class III malocclusion” OR “class III malocclusion” OR “maxillofacial development” OR “angle class II” OR “angle class II malocclusion” OR “skeletal class II malocclusion” OR “class II malocclusion” OR “angle class I” OR “angle class I malocclusion” OR “skeletal class I malocclusion” OR “class I malocclusion”)
LILACS	(“Deglutition Disorders” OR “Deglutition Disorder” OR “Swallowing” OR “swallowed” OR “swallowings” OR “swallowable” OR “swallower” OR “swallowers” OR “swallows” OR “swallow” OR “Swallowing Disorders” OR “Swallowing Disorder” OR “atypical swallowing” OR “atypical deglutition” OR “Myofunctional” OR “trastornos de la deglución” OR “trastorno de la deglución” OR “deglución” OR “deglución atípica” OR “Distúrbios da deglutição” OR “distúrbio da deglutição” OR “deglutição atípica” OR “deglutição” OR “Miofuncional”) AND (“Malocclusions” OR “malocclusion” OR “dental position” OR “dental positions” OR “tooth crowding” OR “crossbite” OR “crossbites” OR “crossbite” OR “crossbites” OR “cross-bite” OR “cross-bites” OR “open bite” OR “openbite” OR “open-bite” OR “Angle’s Classification” OR “Angle Classification” OR “Angles Classification” OR “Angle Class I” OR “Angle Class II” OR “Angle Class III” OR “Class I” OR “Class II” OR “Class III” OR “malocclusion, angle class III” OR “angle class III malocclusion” OR “skeletal class III malocclusion” OR “class III malocclusion” OR “maxillofacial development” OR “angle class II” OR “angle class II malocclusion” OR “skeletal class II malocclusion” OR “class II malocclusion” OR “angle class I” OR “angle class I malocclusion” OR “skeletal class I malocclusion” OR “class I malocclusion” OR “Maloclusiones” OR “maloclusión” OR “posición dental” OR “posiciones dentales” OR “apiñamiento de los dientes” OR “mordida cruzada” OR “mordidas cruzadas” OR “mordida abierta” OR “Clasificación de Angle” OR “Clase I de Angle I” OR “Clase II de Angle” OR “Clase III de Angle” OR “maloclusión, Angle clase III” OR “maloclusión Angle clase III” OR “maloclusión esquelética clase III” OR “maloclusión clase III” OR “desarrollo maxilofacial” OR “clase de Angle II” OR “maloclusión de clase II del Angle” OR “maloclusión esquelética clase II” OR “maloclusión clase II” OR “clase Angle I” OR “maloclusión classe I del Angle” OR “maloclusión clase I esquelética” OR “maloclusión clase I” OR “Maloclusões” OR “maloclusão” OR “má oclusão” OR “posição dentária” OR “posições dentárias” OR “apinhamento dos dentes” OR “mordida cruzada” OR “mordida aberta” OR “mordida profunda” OR “Classificação de angle” OR “Classe I de Angle” OR “Classe II de Angle” OR “Classe III de Angle” OR “Classe I” OR “Classe II” OR “Classe III” OR “má oclusão, classe de Angle III” OR “má oclusão de classe III de Angle” OR “má oclusão esquelética de classe III” OR “má oclusão de classe III” OR “desenvolvimento maxilofacial” OR “classe de Angle II” OR “má oclusão de classe de Angle II” OR “má oclusão esquelética de classe II” OR “má oclusão de classe II” OR “classe de Angle I” OR “má oclusão de classe I de Angle” OR “má oclusão esquelética de classe I” OR “má oclusão de classe I”)
LIVIVO	TI=(“Deglutition Disorders” OR “Deglutition Disorder” OR “Swallowing” OR “swallowed” OR “swallowings” OR “swallowable” OR “swallower” OR “swallowers” OR “swallows” OR “swallow” OR “Swallowing Disorders” OR “Swallowing Disorder” OR “atypical swallowing” OR “atypical deglutition” OR “Myofunctional”) AND TI=(“Malocclusions” OR “malocclusion” OR “dental position” OR “dental positions” OR “tooth crowding” OR “crossbite” OR “crossbites” OR “cross-bite” OR “cross-bites” OR “cross-bite” OR “cross-bites” OR “open-bite” OR “openbite” OR “open-bite” OR “deep bite” OR “Overbites” OR “overbite” OR “deep bite” OR “deep bites” OR “deep bite” OR “deep bites” OR “Over Bite” OR “Over Bites” OR “Angle’s Classification” OR “Angle Classification” OR “Angles Classification” OR “angle class I” OR “angle class II” OR “Angle Class III” OR “Class I” OR “Class II” OR “Class III” OR “malocclusion, angle class III” OR “angle class III malocclusion” OR “skeletal class III malocclusion” OR “class III malocclusion” OR “maxillofacial development” OR “angle class II” OR “angle class II malocclusion” OR “skeletal class II malocclusion” OR “class II malocclusion” OR “angle class I” OR “angle class I malocclusion” OR “skeletal class I malocclusion” OR “class I malocclusion”)
PubMed	(Deglutition Disorders”[MeSH Terms] OR “Deglutition Disorders”[All Fields] OR “Deglutition Disorder”[All Fields] OR “Swallowing”[All Fields] OR “swallowed”[All Fields] OR “swallowings”[All Fields]) OR “swallowable”[All Fields] OR “swallower”[All Fields] OR “swallowers”[All Fields] OR “swallows”[MeSH Terms] OR “swallows”[All Fields] OR “swallow”[All Fields] OR “Swallowing Disorders”[All Fields] OR “Swallowing Disorder”[All Fields] OR “atypical swallowing”[All Fields] OR “atypical deglutition”[All Fields] OR “Myofunctional”[All Fields]) AND (“Malocclusions”[All Fields] OR “malocclusion”[MeSH Terms] OR “malocclusion”[All Fields] OR “dental position”[All Fields] OR “dental positions”[All Fields] OR “tooth crowding”[All Fields] OR “crossbite”[All Fields] OR “crossbites”[All Fields] OR “cross-bite”[All Fields] OR “cross-bites”[All Fields] OR “cross-bite”[All Fields] OR “cross-bites”[All Fields] OR “open-bite”[All Fields] OR “openbite”[All Fields] OR “open-bite”[All Fields] OR “deep bite”[All Fields]) OR “Overbites”[All Fields] OR “overbite”[MeSH Terms] OR “deep bite”[All Fields] OR “deep bites”[All Fields] OR “deep bite”[All Fields] OR “deep bites”[All Fields] OR “Over Bite”[All Fields] OR “Over Bites”[All Fields] OR “Angle’s Classification”[All Fields] OR “Angle Classification”[All Fields] OR “Angles Classification”[All Fields] OR “angle class I”[All Fields] OR “angle class II”[All Fields] OR “Angle Class III”[All Fields] OR “Class I”[All Fields] OR “Class II”[All Fields] OR “Class III”[All Fields] OR “malocclusion, angle class III”[MeSH Terms] OR “angle class III malocclusion”[All Fields] OR “skeletal class III malocclusion”[All Fields] OR “class III malocclusion”[All Fields] OR “maxillofacial development”[All Fields] OR “angle class II”[All Fields] OR “angle class II malocclusion”[All Fields] OR “skeletal class II malocclusion”[All Fields] OR “class II malocclusion”[All Fields] OR “angle class I”[All Fields] OR “angle class I malocclusion”[All Fields] OR “skeletal class I malocclusion”[All Fields] OR “class I malocclusion”[All Fields])
Scopus	(“Deglutition Disorders” OR “Deglutition Disorder” OR “Swallowing” OR “swallowed” OR “swallowings” OR “swallowable” OR “swallower” OR “swallowers” OR “swallows” OR “swallow” OR “Swallowing Disorders” OR “Swallowing Disorder” OR “atypical swallowing” OR “atypical deglutition” OR “Myofunctional”) AND (“Malocclusions” OR “malocclusion” OR “dental position” OR “dental positions” OR “tooth crowding” OR “crossbite” OR “crossbites” OR “cross-bite” OR “cross-bites” OR “cross-bite” OR “cross-bites” OR “open-bite” OR “openbite” OR “open-bite” OR “deep bite” OR “Overbites” OR “overbite” OR “deep bite” OR “deep bites” OR “deep bite” OR “deep bites” OR “Over Bite” OR “Over Bites” OR “Angle’s Classification” OR “Angle Classification” OR “Angles Classification” OR “angle class I” OR “angle class II” OR “Angle Class III” OR “Class I” OR “Class II” OR “Class III” OR “malocclusion, angle class III” OR “angle class III malocclusion” OR “skeletal class III malocclusion” OR “class III malocclusion” OR “maxillofacial development” OR “angle class II” OR “angle class II malocclusion” OR “skeletal class II malocclusion” OR “class II malocclusion” OR “angle class I” OR “angle class I malocclusion” OR “skeletal class I malocclusion” OR “class I malocclusion”)
Web of Science	TS=(“Deglutition Disorders” OR “Deglutition Disorder” OR “Swallowing” OR “swallowed” OR “swallowings” OR “swallowable” OR “swallower” OR “swallowers” OR “swallows” OR “swallow” OR “Swallowing Disorders” OR “Swallowing Disorder” OR “atypical swallowing” OR “atypical deglutition” OR “Myofunctional” AND TS=(“Malocclusions” OR “malocclusion” OR “dental position” OR “dental positions” OR “tooth crowding” OR “crossbite” OR “crossbites” OR “cross-bite” OR “cross-bites” OR “cross-bite” OR “cross-bites” OR “open-bite” OR “openbite” OR “open-bite” OR “deep bite” OR “Overbites” OR “overbite” OR “deep bite” OR “deep bites” OR “deep bite” OR “deep bites” OR “Over Bite” OR “Over Bites” OR “Angle’s Classification” OR “Angle Classification” OR “Angles Classification” OR “angle class I” OR “angle class II” OR “Angle Class III” OR “Class I” OR “Class II” OR “Class III” OR “malocclusion, angle class III” OR “angle class III malocclusion” OR “skeletal class III malocclusion” OR “class III malocclusion” OR “maxillofacial development” OR “angle class II” OR “angle class II malocclusion” OR “skeletal class II malocclusion” OR “class II malocclusion” OR “angle class I” OR “angle class I malocclusion” OR “skeletal class I malocclusion” OR “class I malocclusion”)
Google Scholar	(“atypical swallowing” OR “atypical deglutition”) AND (“Malocclusions”)
Open Grey	(“atypical swallowing” OR “atypical deglutition”) AND (“Malocclusions”)
ProQuest	(“Deglutition Disorders” OR “Deglutition Disorder” OR “Swallowing” OR “swallowed” OR “swallowings” OR “swallowable” OR “swallower” OR “swallowers” OR “swallows” OR “swallow” OR “Swallowing Disorders” OR “Swallowing Disorder” OR “atypical swallowing” OR “atypical deglutition” OR “Myofunctional”) AND (“Malocclusions” OR “malocclusion” OR “dental position” OR “dental positions” OR “tooth crowding” OR “crossbite” OR “crossbites” OR “cross-bite” OR “cross-bites” OR “cross-bite” OR “cross-bites” OR “open-bite” OR “openbite” OR “open-bite” OR “deep bite” OR “Overbites” OR “overbite” OR “deep bite” OR “deep bites” OR “deep bite” OR “deep bites” OR “Over Bite” OR “Over Bites” OR “Angle’s Classification” OR “Angle Classification” OR “Angles Classification” OR “angle class I” OR “angle class II” OR “Angle Class III” OR “Class I” OR “Class II” OR “Class III” OR “malocclusion, angle class III” OR “angle class III malocclusion” OR “skeletal class III malocclusion” OR “class III malocclusion” OR “maxillofacial development” OR “angle class II” OR “angle class II malocclusion” OR “skeletal class II malocclusion” OR “class II malocclusion” OR “angle class I” OR “angle class I malocclusion” OR “skeletal class I malocclusion” OR “class I malocclusion”)

## Appendix 2: Excluded articles and reasons for exclusion (n=76).

[Table t2a]

**Table t2a:**

Author, Year	Reason for exclusion
Alvarez Utria., et al (2016)^1^	4
Aragon de Macedo, P.F. et al (2014)^2^	2
Ardakani, F.E (2006)^3^	2
Baldrighi, S. E. Z. M (1999)^4^	1
Begnoni, G., et al (2020)^5^	2
Bertolini, M. M., et al (2003)^6^	2
Bourdiol, P. et al (2017)^7^	4
Mezzomo C. L., et al (2011)^8^	5
Chiodelli, L., et al (2015)^9^	1
Del Aguila, M. A., et al (2007)^10^	4
Emmerich, A. et al (2004)^11^	3
Farronato, G. P. et al (1982)^12^	6
Genolet, M (1993)^13^	5
Grabowski R, et al (2007)^14^	4
Gustafsson M, et al (1975)^15^	4
Haynes, S. (1975)^16^	4
Ichida, t. et al (1999) ^17^	4
Jin, I. J.; Yang, W. S. (1987)^18^	4
Leme, M. S. et al (2013)^19^	4
Limme, M. (1991)^20^	4
Lin, L. H. et al (2013) ^21^	5
Lindsey, C. A.; English, J. D. (2003)^22^	5
Lopes C. M. I.; Barros, A. M. S. (2019)^23^	2
Lopes Freire, G. M.;(2016) ^24^	4
Lyszczarz, J. et al (2012)^25^	4
MacAvoy, S. K. et al (2016) ^26^	4
Machado Jr, A. J.; Crespo, A. N. (2012)^27^	2
Machado Jr, A. J.; Crespo, A. N. (2012)^28^	2
Machado Jr, A. J.; Crespo, A. N. (2010)^29^	2
Maciel, C. T.; Leite, I. C. (2005)^30^	4
Maciel, C. T., et al (2006)^31^	4
Marcomini, L., et al (2010)^32^	3
Martin, C. et al (2012) ^33^	4
Mason, R. M. (2011)^34^	5
Medeiros, A. P. M. et al (2009)^35^	4
Morari. A. c. et al (2019)^36^	4
Mutlu, E. et al (2019)^37^	4
Nashashibi, I. A. (1987)^38^	4
Ngom, P. I. et al (2007)^39^	4
Nihi, V. S. et al (2015)^40^	4
Ning, B. et al (2007)^41^	4
Ono, T. et al (1998)^42^	4
Onyeaso, C.O. et al (2008)^43^	4
Gaymer, G.O. et al (1971)^44^	1
Ovsenik, M., et al (2007)^45^	3
Owens, S. et al (2002) ^46^	4
Padovan, B. A. (1995) ^47^	5
Parisella, V. D et al (2002) ^48^	1
Perkins, J. A. ^49^	5
Piancino, M. G. (2012)^50^	4
Picinato, M. et al (2012)^51^	1
Premkumar, S. et al (2011)^52^	4
Primozic, J. et al (2013)^53^	4
Regalo, S. C. H. et al (2003)^54^	4
Regina, C. et al (2005)^55^	2
Rochelle, I. M. F. et al (2010)^56^	3
Saccomanno, S. et al (2012) ^57^	1
Schneider, E. et al. (1975) ^58^	1
Seemann, J et al (2011) ^59^	3
Shenoy, U. et al (2015) ^60^	4
Silva, C. E. F. M et al (1994)^61^	3
Silva, M. et al (2014)^62^	5
Silva, R. A. (2016)^63^	3
Sorokin, A. et al (2015)^64^	1
Stormer, K. et al (1999)^65^	2
Taner, T. et al (2013)^66^	5
Truer. U. et al (1986)^67^	3
Tosello, D. O. (1999)^68^	2
Trannin, P. G. et al (2012)^69^	4
Turvey, T. A. et al (1976)^70^	1
Urzal, V. et al (2013)^71^	4
Volk, J. et al (2010) ^72^	2
Williamson, E. et al (1990)^73^	1
Xu, K. et al (2016)^74^	1
Xue, M. et al (2009)^75^	4
Zhou, Y. et al (1995)^76^	2

APPENDIX 2 REFERENCES

1. Alvarez Utria Y, González Rodríguez Y, Ureña Espinosa M, Rodríguez González Y. Prevalence of oral deforming habits in students between the ages of six to nine. Rev Electron Dr. Zoilo E. Marinello Vidaurreta. 2016;41(8).

2. Macedo PF, Bianchini EM. Myofunctional orofacial examination: comparative analysis in young adults with and without complaints. CoDAS. 2014;26:464-70.

3. Ardakani FE. Evaluation of swallowing patterns of the tongue using real-time B-mode sonography. J Contemp Dent Pract. 2006 Jul 1;7(3):67-74.

4. Baldrighi SEZdM. Alterações neuromusculares associadas à atresia do arco dentário superior e consequentes à expansão rápida da maxila: estudo longitudinal. (Thesis) Universidade Federal de São Paulo.1999:119p. http://repositorio.unifesp.br/handle/11600/16270 (Accessed December 2020).

5. Begnoni G, Cadenas de Llano-Pérula M, Dellavia C, Willems G. Cephalometric traits in children and adolescents with and without atypical swallowing: A retrospective study. Eur J Paediatr Dent. 2020 Mar;21(1):46-52.

6. Bertolini MM, Vilhegas S, Norato DY, Paschoal JR. Cephalometric evaluation in children presenting adapted swallowing during mixed dentition. Int J Orofacial Myology. 2003 Nov;29:29-41.

7. Bourdiol P, Soulier-Peigue D, Lachaze P, Nicolas E, Woda A, Hennequin M. Only severe malocclusion correlates with mastication deficiency. Arch Oral Biol. 2017 Mar;75:14-20.

8. Mezzomo CL, Machado PG, Pacheco AdB, Gonçalves BFT, Hoffmann CF. The implications of class II angle and class II type skeletal disproportion on the myofunctional aspect. Rev CEFAC. 2011;13(4):728-34.

9. Chiodelli L, Pacheco AdB, Missau TS, Silva AMTd, Corrêa ECR. Association among stomatognathic functions, dental occlusion and temporomandibular disorder signs in asymptomatic women. Rev CEFAC. 2015;17(1):117-25.

10. Del Águila Ochoa MA, Céspedes Porras J. Relation between the atypical swallowing and opened bite with the presence of dislalias in the children of six to ten years old of Educative Center Nuestra Señora del Consuelo. Kiru. 2007;4(1):20-3.

11. Emmerich A, Fonseca L, Elias AM, de Medeiros UV. The relationship between oral habits, oronasopharyngeal alterations, and malocclusion in preschool children in Vitória, Espírito Santo, Brazil. Cad Saúde Pública. 2004 May-Jun;20(3):689-9.

12. Farronato GP, Preteroti AM, Salvato A, Bruno E. Relation between skeletal open bite and atypical deglutition. Arch Stomatol (Napoli). 1982 Jan-Mar;23(1):53-74.

13. Genolet M. Description study about the relation in the facial patterns with the atypical deglutition and with the anterior open bite. (Thesis) Universidad Nacional de Rosario. 1993:182p. (accessed December 2020).

14. Grabowski R, Stahl F, Gaebel M, Kundt G. Relationship between occlusal findings and orofacial myofunctional status in primary and mixed dentition: Part I: Prevalence of Malocclusions. J Orofac Orthop. 2007 Jan;68(1):26-37.

15. Gustafsson M, Ahlgren J. Mentalis and orbicularis oris activity in children with incompetent lips. An electromyographic and cephalometric study. Acta Odontol. Scand. 1975;33(1):355-63.

16. Haynes S. A study of overjet values and their relationship to the type of swallow and type of lip activity during swallowing. Br J Orthod. 1975 Apr;2(2):69-72.

17. Ichida T, Takiguchi R, Yamada K. Relationship between the lingual-palatal contact duration associated with swallowing and maxillofacial morphology with the use of electropalatography. Am J Orthod Dentofacial Orthop. 1999 Aug;116(2):146-51.

18. Jin IJ, Yang WS. A cinefluoroscopic study of oropharyngeal movement of the Class III malocclusion patients during swallowing. Korean J Orthod. 1987 Mar;17(1):119-134.

19. Leme MS, Barbosa TD, Gaviao MBD. Relationship among oral habits, orofacial function and oral health-related quality of life in children. Braz Oral Res. 2013 May-Jun;27(3):272-8.

20. Limme M. Orthodontic consequences of mouth-breathing. Rev Belge Med Dent (1984). 1991;46(4):39-50.

21. Lin LH, Huang GW, Chen CS. Etiology and treatment modalities of anterior open bite malocclusion. JECM. 2013;5(1):1-4.

22. Lindsey CA, English JD. Orthodontic treatment and masticatory muscle exercises to correct a Class I open bite in an adult patient. Am J Orthod Dentofacial Orthop. 2003 Jul;124(1):91-8.

23. Lopes CMI, Barros AMdS. Presença de mordida aberta anterior na dentadura mista relação com hábitos bucais deletérios. Ortho Sci, Orthod Sci Pract. 12(46):82-9.

24. Lopes Freire GM, Espasa Suarez de Deza JE, Rodrigues da Silva IC, Butini Oliveira L, Ustrell Torrent JM, Boj Quesada JR. Non-nutritive sucking habits and their effects on the occlusion in the deciduous dentition in children. Eur J Paediatr Den. Dec 2016, 17(4):301-6.

25. Łyszczarz J, Szot W, Loster BW. Relation between oral breathing and the frequency of malocclusions and respiratory efficiency in adolescence. J Stoma. 2012;65:714-28.

26. MacAvoy SK, Jack HC, Kieser J, Farella M. Effect of occlusal vertical dimension on swallowing patterns and perioral electromyographic activity.J Oral Rehabil. 2006;43(7):481−7.

27. Machado Jr AJ, Crespo AN. Radiographic position of the hyoid bone in children with atypical deglutition. Eur J Orthod. 2012 Feb;34(1):83-7.

28. Machado Júnior AJ, Crespo AN. Cephalometric evaluation of the oropharyngeal space in children with atypical deglutition. Braz J Otorhinolaryngol. 2012 Feb;78(1):120-5.

29. Machado Jr AJ, Crespo AN. A lateral cephalometric x-ray study of selected vertical dimensions in children with atypical deglutition. Int J Orofacial Myology. 2010 Nov;36:17-26.

30. Maciel CT, Leite IC. Etiological aspects of anterior open bite and its implications to the oral functions. Pro Fono. 2005 Apr-Dec;17(3):293-302.

31. Maciel CTV, Barbosa MH, Toldo CdA, Faza FCB, Chiappetta ALdML. Orofacial dysfunctions in pacient under orthodontic treatment. Rev CEFAC. 2006;8(4):456-66.

32. Marcomini L, Santamaria Jr M, Lucato AS, Santos JCBd, Tubel CAM. Prevalence of malocclusion and its relationship with functional changes in the breathing and in the swallowing. Braz Dent Sci. 2010;13:52-58.

33. Martin C, Palma JC, Alaman JM, Lopez-Quinones JM, Alarcon JA. Longitudinal evaluation of sEMG of masticatory muscles and kinematics of mandible changes in children treated for unilateral cross-bite. J Electromyogr Kinesiol. 2012 Aug;22(4):620-8.

34. Mason RM. Myths that persist about orofacial myology. Int J Orofacial Myology. 2011 Nov;37:26-38.

35. Medeiros APM, Ferreira JTL, de Felício CM. Correlation between feeding methods, non-nutritive sucking and orofacial behaviors. Pro Fono. 2009 Oct-Dec;21(4):315-9.

36. Morari AC, Santos PRd, Nabarrette M, et al. Deglutition pattern in Angle’s Class II malocclusion. Rev CEFAC. 2019;21(2):e11818.

37. Mutlu E, Parlak B, Kuru S, Oztas E, Pinar-Erdem A, Elif E. Evaluation of crossbites in relation with dental arch widths, occlusion type, nutritive and non-nutritive sucking habits and respiratory factors in the early mixed dentition. Oral Health Prev Dent. 2019;17(5):447-55.

38. Nashashibi IA. Variation of swallowing patterns with malocclusions. J Pedod. 1987 Summer;11(4):332-7.

39. Ngom PI, Diagne F, Aïdara-Tamba AW, Sene A. Relationship between orthodontic anomalies and masticatory function in adults. Am J Orthod Dentofacial Orthop. 2007 Feb;131(2):216-22.

40. Nihi VS, Maciel SM, Jarrus ME, et al. Pacifier-sucking habit duration and frequency on occlusal and myofunctional alterations in preschool children. Braz Oral Res. 2015;29(1):1-7.

41. Ning B, He W, Wei LY, Xiang DY, Pang XN. Swallowing with the skeletal class III malocclusion before and after the treatment on M-mode ultrasonography. CJMIT. 2007;23(10):1465-8.

42. Ono T, Hiyama S, Tsuiki S, et al. Tongue movement and genioglossus muscle activity during swallowing in anterior open bite. J Dent Res. 1998;77:1015-1015.

43. Onyeaso CO, Isiekwe MC. Oral habits in the primary and mixed dentitions of some nigerian children: A Longitudinal study. Oral Health Prev Dent. 2008;6(3):185-90.

44. Otero Gaymer G, Otero Martinez J, Otero Martinez H. Dynamics of swallowing and orthodontics. Revista de la Federacion Odontologica Ecuatoriana. 1971;1:381-385.

45. Ovsenik M, Farcnik FM, Korpar M, Verdenik I. Follow-up study of functional and morphological malocclusion trait changes from 3 to 12 years of age. Eur J Orthod. 2007 Oct;29(5):523-9.

46. Owens S, Buschang PH, Throckmorton GS, Palmer L, English J. Masticatory performance and areas of occlusal contact and near contact in subjects with normal occlusion and malocclusion. Am J Orthod Dentofacial Orthop. 2002 Jun;121(6):602-9.

47. Padovan BA. Neurofunctional reorganization in myo-osteo-dentofacial disorders: complementary roles of orthodontics, speech and myofunctional therapy. Int J Orofacial Myology. 1995 Nov;21:33-40.

48. Parisella V, Vozza I, Capasso F, et al. Cephalometric evaluation of the hyoid triangle before and after maxillary rapid expansion in patients with skeletal class II, mixed dentition, and infantile swallowing. Ann Stomatol (Roma). 2012 Jul;3(3-4):95-9.

49. Perkins JA. Overview of macroglossia and its treatment. Curr Opin Otolaryngol Head Neck Surg. 2009 Dec;17(6):460-5.

50. Piancino MG, Isola G, Merlo A, Dalessandri D, Debernardi C, Bracco P. Chewing pattern and muscular activation in open bite patients. J Electromyogr Kinesiol. 2012 Apr;22(2):273-9.

51. Picinato-Pirola M, Silva J, Mestriner- Junior W, Mello FF, Trawitzki L. Relation between masticatory efficiency and bite force in dentofacial deformities. JOMS. 2012;70:e-64.

52. Premkumar S, Venkatesan SA, Rangachari S. Altered oral sensory perception in tongue thrusters with an anterior open bite. Eur J Orthod. 2011 Apr;33(2):139-42.

53. Primožič J, Franchi L, Perinetti G, Richmond S, Ovsenik M. Influence of sucking habits and breathing pattern on palatal constriction in unilateral posterior crossbite-a controlled study. Eur J Orthod. 2013;35(5):706-12.

54. Regalo SCH, Vitti M, Hallak JEC, et al. EMG analysis of the upper and lower fascicles of the orbicularis oris muscle in deaf individuals. Electromyogr Clin Neurophysiol. 2003 Sep;43(6):367-72.

55. Regina C, Katz T, Rosenblatt A. Nonnutritive sucking habits and anterior open bite in Brazilian children: A longitudinal study. Pediatr Dent. 2005 Sep-Oct;27(5):369-73.

56. Rochelle IMF, Tagliaferro EPS, Pereira AC, Meneghim MC, Nóbilo KA, Bovi Ambrosano GM. Breastfeeding, deleterious oral habits and malocclusion in 5-year-old children in São Pedro, SP, Brazil. Dental Press J Orthod. 2010;15(2):71-81.

57. Saccomanno S, Antonini G, D’Alatri L, D’Angelantonio M, Fiorita A, Deli R. Causal relationship between malocclusion and oral muscles dysfunction: a model of approach. Eur J Paediatr Dent 2012;13(4):321-3.

58. Schneider E, Schmidt H, Rakosi T. Die Korrelation zwischen dem Zungenpressen und dem Aufbau des Gesichtsschädels. [Correlation between tongue pressure and structure of the facial bones]. Fortschritte der Kieferorthopadie. 1975;36:379-390.

59. Seemann J, Kundt G, Stahl de Castrillon F. Relationship between occlusal findings and orofacial myofunctional status in primary and mixed dentition: part IV: interrelation between space conditions and orofacial dysfunctions. J Orofac Orthop. 2011 Mar;72(1):21-32.

60. Shenoy U, Hazarey P, Jakhare P, Mute BK. Cephalometric appraisal of tongue and related soft tissues in normal and open bite subjects at rest.J Clin Diagn Res. 2015 Jan; 9(1): ZC16-ZC20.

61. Silva CEFdM, Silva MFCdO, Ruela RdS, Pardini LC. Prevalência da deglutição atípica em jovens adultos universitário. Rev Fac Odontol Lins. 1994;6(20):27-30.

62. Silva M, Manton D. Oral habits-part 2: beyond nutritive and non-nutritive sucking. J Dent Child. 2014;81(3):140-6.

63. Silva RA, Jóias RM, Josgrilberg E, Rode SM, Paranhos LR, Jóias RP. The correlation between malocclusions and morphofunctional aspects: analysis of patients aged from 7 to 12 years old. Braz Dent Sci. 2016;19(4):90-7.

64. Sorokin A, Cassir N, Desplats E, Huynh N. The impact of orofacial myofunctional therapy on the reestablishment of nasal breathing and the stability of orthodontic treatment. tongue thrust: To treat or not to treat? Cleft Palate-Craniofacial Journal. 2015;52:e125-e126.

65. Störmer K, Pancherz H. Electromyography of the perioral and masticatory muscles in orthodontic patients with atypical swallowing. J Orofac Orthop. 1999;60(1):13-23.

66. Taner T, Saglam-Aydinatay B. Physiologic and dentofacial effects of mouth breathing compared to nasal breathing. Nasal Physiology and Pathophysiology of Nasal Disorders. 2013:567-588.

67. Thüer U, Ingervall B. Pressure from the lips on the teeth and malocclusion. Am J Orthod Dentofacial Orthop. 1986 Sep;90(3):234-42.

68. Tosello DO, Vitti M, Berzin F. EMG activity of the orbicularis oris and mentalis muscles in children with malocclusion, incompetent lips and atypical swallowing--part II. J Oral Rehabil. 1999;26:644-649.

69. Trannin PG, Maffei C, Azevedo-Alanis LR, Camargo ES, Vianna-Lara MS. Masticatory function characteristics in individuals with unilateral posterior crossbite. Arch Oral Res. 2012;8:127-132.

70. Turvey TA, Journot V, Epker BN. Correction of anterior open bite deformity: A study of tongue function, speech changes, and stability. J Maxillofac Surg. 1976 Jun;4(2):93-101.

71. Urzal V, Braga AC, Ferreira AP. The prevalence of anterior open bite in Portuguese children during deciduous and mixed dentition-Correlations for a prevention strategy. Int Orthod. 2013; 11: 93-103.

72. Volk J, Kadivec M, Mušič MM, Ovsenik M. Three-dimensional ultrasound diagnostics of tongue posture in children with unilateral posterior crossbite. Am J Orthod Dentofacial Orthop. 2010 Nov;138(5):608-12.

73. Williamson EH, Hall JT, Zwemer JD. Swallowing patterns in human subjects with and without temporomandibular dysfunction. Am J Orthod Dentofacial Orthop 1990;98:507-11.

74. Xu K, Zeng J, Xu T. Effect of an intraoral appliance on tongue pressure measured by force exerted during swallowing. Am J Orthod Dentofacial Orthop. 2016 Jan;149(1):55-61.

75. Xue M, Gao XH, Pang XN, Bai YX. [Ultrasonographic evaluation of tongue movement during swallowing in severe skeletal Class III malocclusion in adult patients]. Zhonghua Kou Qiang Yi Xue Za Zhi 2009;44(5):289-92.

76. Zhou Y, Fu M, Wang C. [Swallowing pattern in skeletal Class III malocclusion]. Zhonghua Kou Qiang Yi Xue Za Zhi 1995;30(6):355-8, 384.
